# Bilateral Renal Vein Thrombosis and Chylous Ascites in Phospholipase A2 Receptor-Associated Membranous Nephropathy: A Case Report

**DOI:** 10.7759/cureus.63434

**Published:** 2024-06-29

**Authors:** Kazushige Shiraishi, FNU Chesta, Yoshito Nishimura, Christina M Chong

**Affiliations:** 1 Internal Medicine, John A. Burns School of Medicine, University of Hawaii at Manoa, Honolulu, USA

**Keywords:** renal vein thrombosis, nephrotic syndrome, pla2r, membranous nephropathy, chylous ascites

## Abstract

Phospholipase A2 receptor (PLA2R)-associated membranous nephropathy is an important cause of nephrotic syndrome that can lead to a variety of systemic manifestations. Chylous ascites and bilateral renal vein thrombosis are rare manifestations in adult nephrotic syndrome, and there have been no reported cases demonstrating both chylous ascites and bilateral renal vein thrombosis in patients with PLA2R-associated membranous nephropathy. Here, we report the first case of PLA2R-associated membranous nephropathy complicated by renal vein thrombosis and chylous ascites successfully treated with anticoagulation and rituximab.

A 65-year-old African American male presented with abdominal pain for four days, hematochezia for one day, and lower extremity edema for one year. Blood pressure was 158/73 mmHg and other vital signs were normal. Physical examination revealed abdominal distention, periumbilical tenderness, and bilateral lower extremity edema. Laboratory analysis showed high serum creatinine, hypoalbuminemia, hyperlipidemia, and proteinuria on 24-hour urine chemistry, all consistent with nephrotic syndrome. Abdominal computed tomography scan demonstrated nonocclusive bilateral renal vein thrombosis with ascites. Paracentesis revealed chylous ascites. Continuous heparin infusion was started for thrombosis. Esophagoduodenoscopy and colonoscopy did not reveal a source of bleeding. Serum anti-PLA2R was found positive, suggesting membranous nephropathy. Rituximab, along with warfarin switched from heparin, successfully controlled disease activity.

Chylous ascites in nephrotic syndrome is thought to be associated with bowel edema. In our case, we hypothesize that renal vein thrombosis caused lymphatic fluid leakage by increasing lymphatic pressure. The case illustrates the importance of considering membranous nephropathy as a cause of chylous ascites and renal vein thrombosis. Development of lymphatic imaging techniques is warranted to clarify the pathophysiology.

## Introduction

Phospholipase A2 receptor (PLA2R)-associated membranous nephropathy is one of the most common causes of nephrotic syndrome, resulting in diverse systemic manifestations [1]. Chylous ascites and renal vein thrombosis are rare complications of nephrotic syndrome [2], and their pathophysiology in the context of nephrotic syndrome is unclear. There have been no cases showing chylous ascites and bilateral renal vein thrombosis in a patient with PLA2R-associated membranous nephropathy. Herein, we present the first case of chylous ascites and bilateral renal vein thrombosis secondary to nephrotic syndrome due to PLA2R-associated membranous nephropathy successfully treated with anticoagulation and rituximab.

## Case presentation

A 65-year-old African American male with a history of hypertension and peripheral artery disease presented with acute-onset abdominal pain for four days, hematochezia for one day, and worsening lower extremity edema up to the thigh for one year. One year prior to admission, the patient noticed bilateral lower extremity edema which extended to his thighs five months prior to admission. Four days prior to admission, the patient noticed abdominal distention then developed acute onset abdominal pain and hematochezia.

Physical examination revealed a well-nourished male in no distress with a blood pressure of 158/73 mmHg and normal temperature, pulse, and respirations. Cardiac and pulmonary examinations were normal. The abdomen was distended with periumbilical tenderness and normal bowel sounds without rebound or guarding. Shifting dullness was not clear. Bilateral pitting edema extending to the thighs was noted. Rectal examination showed bright red blood. Laboratory analysis (Table 1) was remarkable for hemoglobin 10.3 g/dL and findings suggesting nephrotic syndrome (blood urea nitrogen 15 mg/dL, creatinine 1.9 mg/dL [baseline 1.1 mg/dL, six years prior to admission], albumin 2.0 g/dL, total protein 5.0 g/dL, cholesterol 222 mg/dL, and triglycerides 245 mg/dL), with significant proteinuria of 6452 mg on 24-hour urine protein collection.

**Table 1:** Laboratory data WBC, white blood cell; BUN, blood urea nitrogen; AST, aspartate transaminase; ALT, alanine transaminase; ALP, alkaline phosphatase; LDH, lactate dehydrogenase; PT-INR, prothrombin time-international normalized ratio; ALT, alanine transaminase; ALP, alkaline phosphatase; LDH, lactate dehydrogenase; PT-INR, prothrombin time-international normalized ratio; HDL, high-density lipoprotein; PLA2R, phospholipase A2 receptor; ANA, antinuclear antibody; ANCA, antineutrophilic cytoplasmic antibody; HBs, hepatitis B surface; HBc, hepatitis B core; HCV, hepatitis C virus; C3, complement component 3; C4, complement component 4; SPEP, serum protein electrophoresis; UPEP, urine protein electrophoresis.

Table 1. Laboratory data				
WBC	11.70 x 10^3^/μL		Total cholesterol	222 mg/dL
Hemoglobin	10.3 g/dL		HDL cholesterol	41 mg/dL
Hematocrit	29.70%		Triglycerides	245 mg/dL
Platelet	315 x 10^3^/μL		Urine protein	(4+)
Na	141 mEq/L			6.4 g/day
K	2.9 mEq/L		Urine blood	Mod
Cl	115 mEq/L		Anti-PLA2R Antibody	1:2560
Glucose	112 mg/dL		ANA	<40
BUN	15 mg/dL		ANCA	Negative
Creatinine	1.9 mg/dL		HBs Antigen	Negative
Total protein	5.0 g/dL		Anti-HBs Antibody	Positive
Albumin	2.0 g/dL		Anti-HBc Antibody	Negative
AST (SGOT)	17 IU/L		Anti-HCV Antibody	Negative
ALT (SGPT)	11 IU/L		Rapid Plasma Reagin	Non-reactive
ALP	118 IU/L		C3	119 mg/dL
Total Bilirubin	<0.2 mg/dL		C4	46 mg/dL
LDH	329 IU/L		SPEP	No monoclonal protein
PT-INR	1.1		UPEP	No monoclonal protein

**Figure 1:**
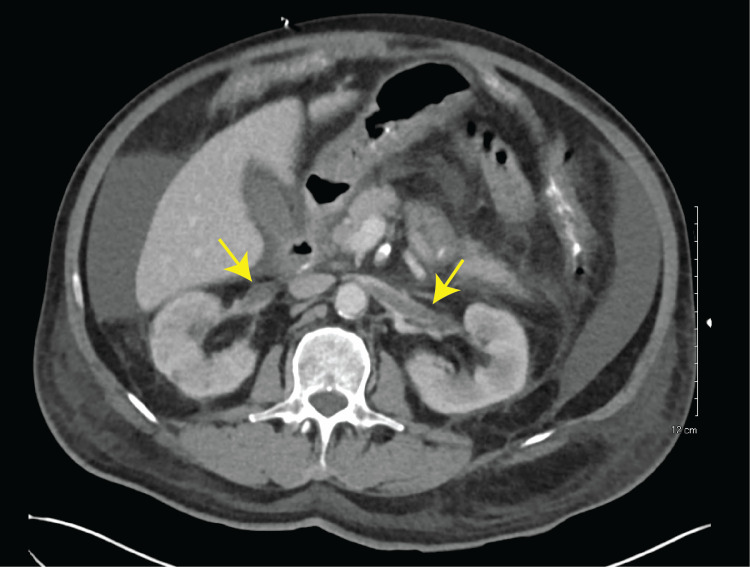
Abdominal CT scan with contrast revealed bilateral renal vein thrombosis and ascites Abdominal CT scan with contrast showed bilateral renal vein thrombosis. Yellow arrows indicate renal vein thrombosis.

**Figure 2:**
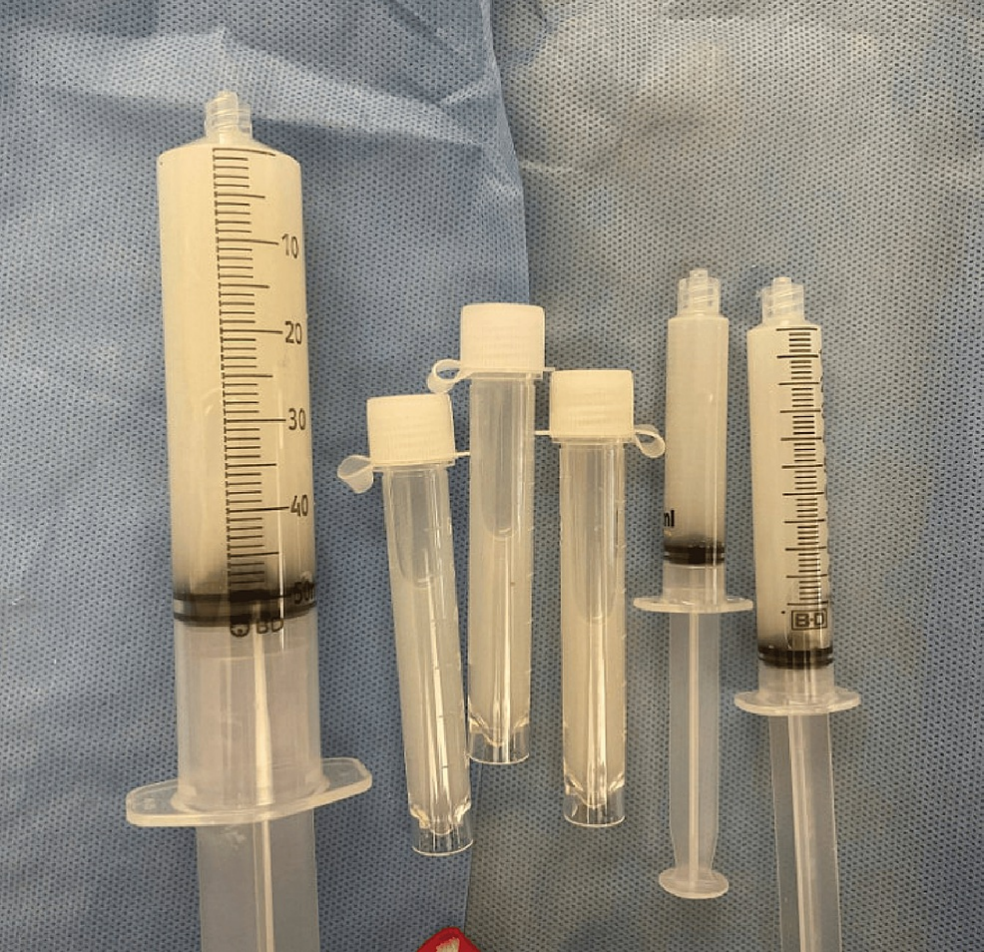
Chylous ascites obtained from paracentesis Picture showing milky white chylous ascites obtained by paracentesis performed on the day of admission.

Abdominal contrast computed tomography (CT) showed non-occlusive bilateral renal vein thrombosis with moderate amount of ascites without other significant findings (Figure 1). Paracentesis was performed for his ascites to exclude the possibility of spontaneous bacterial peritonitis. Ascitic fluid was milky white (Figure 2), suggesting chylous ascites. Ascitic chemistry analysis revealed low cholesterol (10 mg/dL) and high triglycerides (131 mg/dL). Ascitic fluid culture was negative.

On admission, the patient was started on an intravenous continuous heparin infusion for the treatment of bilateral renal vein thrombosis. On day three, the patient underwent esophagoduodenoscopy and colonoscopy to evaluate for any source of bleeding for his hematochezia and for malignancy given nephrotic syndrome. The studies showed few localized diminutive erosions with no bleeding and no stigmata of recent bleeding in the stomach and non-bleeding external and internal hemorrhoids, but were negative for detecting an active source of bleeding or malignancy. He did not have further signs of gastrointestinal bleeding, and the cause of hematochezia was thought to be secondary to gastric erosions or hemorrhoids.

Initial workup for nephrotic syndrome was positive for anti-PLA2R antibody titer at 1:2560. Additional negative workup included: antinuclear antibody (ANA); anti-neutrophil cytoplasmic antibody (ANCA); hepatitis B and C infection screening; HIV infection; rapid plasma reagin; and monoclonal protein from serum and urine protein electrophoresis (Table 1). Complement components 3 and 4 levels were not decreased. Kidney biopsy was offered but the patient opted out due to bleeding risk. Following Kidney Disease Improving Global Outcomes (KDIGO) clinical practice guidelines, the patient was diagnosed with membranous nephropathy based on anti-PLA2R antibody levels [3]. Chest CT was negative for malignancy that could explain his membranous nephropathy. The patient was considered at high risk for progressive loss of kidney function [3], and rituximab was started during the hospital stay, which successfully controlled the disease activity. The patient was eventually discharged on warfarin, torsemide, and lisinopril with nephrology follow-up. There was no ascites on physical examination one month after discharge.

## Discussion

To our knowledge, this is the first case of PLA2R-associated membranous nephropathy complicated by bilateral renal vein thrombosis and chylous ascites successfully treated with anticoagulation and rituximab. The prevalence, characteristics, and management of chylous ascites in nephrotic syndrome remain unclear [4]. This uncertainty is likely because clinicians often do not perform paracentesis when ascites is present in patients with nephrotic syndrome, as they typically attribute the cause of ascites to third spacing. However, due to the possibility of concurrent infection or malignancy, paracentesis should be performed in these cases. Low ascitic cholesterol levels (10-62 mg/dL) and high ascitic triglycerides (90-529 mg/dL) with a triglyceride to cholesterol ratio greater than 7.0 were previously reported as good indicators for the diagnosis of chylous ascites in nephrotic syndrome [5]. Our patient’s laboratory findings were all consistent with these numbers. 

The etiology of chylous ascites in nephrotic syndrome is unclear. Some studies attribute the causes to possible bowel edema induced by hypoalbuminemia [5-7]. Our case suggests a possible association between bilateral renal thrombosis and chylous ascites. A tempting hypothesis is that renal thrombosis increases renal lymphatic pressure, leading to leakage of lymphatic fluid into the peritoneal space. Similar pathophysiology of chylothorax is seen in patients with central venous thrombosis [8].

The renal lymphatic system has been suggested to act as a "safety valve" to protect the kidney from elevated intrarenal pressures in the setting of renal vein thrombosis [9]. This has been shown experimentally by occluding the renal vein in animals, causing a dramatic increase in lymphatic pressure as measured by catheterization of the renal capsule lymphatic vessel [10]. There are technical limitations to understanding the pathophysiology of chylous ascites in nephrotic syndrome because renal lymphatics cannot be visualized in humans with existing methods [9]. The invention of further imaging techniques, including injection of the probe into the renal interstitium, may be needed to elucidate the fundamental mechanisms of chylous ascites in nephrotic syndrome.

## Conclusions

We are reporting a case of PLA2R-associated membranous nephropathy complicated by chylous ascites and bilateral renal vein thrombosis that was effectively treated with anticoagulation and rituximab. The effective treatment of our patient with both anticoagulation and rituximab highlights the potential efficacy of this treatment strategy in similar cases. Our case also raises interesting questions about the underlying pathophysiology linking membranous nephropathy, chylous ascites, and renal vein thrombosis. Further research is warranted to establish evidence-based treatment strategies for patients with PLA2R-associated membranous nephropathy with chylous ascites and renal vein thrombosis.
